# Robustness and Vulnerability of Networks with Dynamical Dependency Groups

**DOI:** 10.1038/srep37749

**Published:** 2016-11-28

**Authors:** Ya-Nan Bai, Ning Huang, Lei Wang, Zhi-Xi Wu

**Affiliations:** 1 School of Reliability and Systems Engineering, Beihang University, Beijing, 100191, P.R. China; 2 Key Laboratory of Science & Technology on Reliability & Environmental Engineering, Beihang University, Beijing, 100191, P.R. China; 3 School of Automation Science and Electrical Engineering, Beihang University, Beijing, 100191, P.R. China; 4 Institute of Computational Physics and Complex Systems, Lanzhou University, Lanzhou, Gansu, 730000, P.R. China

## Abstract

The dependency property and self-recovery of failure nodes both have great effects on the robustness of networks during the cascading process. Existing investigations focused mainly on the failure mechanism of static dependency groups without considering the time-dependency of interdependent nodes and the recovery mechanism in reality. In this study, we present an evolving network model consisting of failure mechanisms and a recovery mechanism to explore network robustness, where the dependency relations among nodes vary over time. Based on generating function techniques, we provide an analytical framework for random networks with arbitrary degree distribution. In particular, we theoretically find that an abrupt percolation transition exists corresponding to the dynamical dependency groups for a wide range of topologies after initial random removal. Moreover, when the abrupt transition point is above the failure threshold of dependency groups, the evolving network with the larger dependency groups is more vulnerable; when below it, the larger dependency groups make the network more robust. Numerical simulations employing the Erdős-Rényi network and Barabási-Albert scale free network are performed to validate our theoretical results.

Complex networks are increasingly being investigated in various fields of nature and society^1^,^2^,^3^,^4^,^5^ and from different angles, such as collective behaviour^6^,^7^,^8^,^9^,^10^, robustness^11^,^12^, controllability^13^,^14^, etc. Many findings have been revealed through the discovery of statistical and topological properties of complex networks or via modelling and analysing network behaviours (or characteristics). All of these results can help us well explain distinct phenomena of complex networks in some ways. However, more challenges remain to fully understand complex networks. Cascading failure is one of the fascinating and significant challenges.

Large cascades are common in most complex networks^15^. Earlier works regarding the cascading process in complex networks focused on failures triggered just by the removal of a single node^16^ or small initial shocks^17^. Recently, the dependency property, modelled by dependency links^18^,^19^,^20^, has been proposed to study the effects of dependency among nodes on the evolution of complex networks, especially for cascading failures. One of the characteristics discriminating them from the traditional connectivity links is that dependent links present the relations among local nodes, not the global topological relations of a network. According to ref. 18, dependency studies usually focus either on failures due to overloads in networks or on local dependency models in which each node’s state depends only on the states of its neighbours and therefore a failing node will also cause its neighbours to fail, etc. After dependency groups were proposed, many researchers expanded these studies to random dependency models. One fundamental difference between local dependency models and random dependency models is that dependencies between nodes are either confined to adjacent nodes or not.

In dependency models, network nodes depend on each other, forming a dependency group. If any node in the dependency group fails, the group will fail totally, i.e., all of the other nodes of this group will fail^18^. For example, in financial networks^18^, the trading and sale connections between companies can be abstracted as the connectivity links of networks and companies in the same industrial chain could form a dependency group. If one company fails, all other companies in the same industrial chain also fail due to the rupture of the industrial chain. Another example is online social networks (Facebook or Twitter)^18^: Each individual communicates with his friends. These communication relationships are defined as connectivity links, thus forming social networks through which information and rumours can spread. It is noted that many individuals will only participate in a social network if other individuals with common interests also participate in that social network. These particular relationships are denoted by dependency links. Concerning the dependency property, Parshani *et al*.^18^ proposed an analytical model to study the robustness of networks that include both connectivity and dependency links and found, surprisingly, that a broader degree distribution increases the vulnerability of these networks to random failures, which is opposite to how networks containing only connectivity links behave. The dependency link in ref. 18 represents a static dependency relation between two nodes. Apparently, this assumption, i.e., that only a pair of nodes depends on each other, seems quite unreasonable; Bashan *et al*.^21^ theoretically analysed the effects of different distributions of dependency links on network robustness. For asymmetric dependency based on degree, Li *et al*.^22^ showed that an asymmetric dependency makes networks more robust than a symmetric one and that the percolation transition point is not sensitive to the number of asymmetric dependency nodes. Because of the situation in real networks in which one failed node may not always break the functionality of a dependency group, Wang *et al*.^23^ studied a conditional cascading failure model in which a dependency group fails only when more than a fraction of nodes fails. They found that the network becomes more robust as the fraction increases. Moreover, networks with dependency groups or links have also been studied in the form of interdependent networks and multilayer networks, also showing the fragility of networks when nodes depend on each other^20^,^24^. Among these efforts, some new phenomena have also been found, e.g., assortativity^25^ and coupling strength^26^ decrease the robustness of interdependent networks, intersimilarity^27^ and short dependency distances^28^ have considerable effects on reducing the cascading failures, and percolation transitions are not always sharpened by making networks interdependent^29^.

From previous discussions, static dependency is a common assumption when investigating the dependency property. One key reason is that the dependency relations among nodes are considered to be fixed during the process of cascading failure, which simplifies the theoretical analysis of cascading failures tactfully regarding the dependency property. Yet, it seems more appropriate to introduce dynamical dependency groups to network evolution partially due to the ubiquitousness of the dynamical dependency property in real networks. For instance, acquisitions and mergers have easily taken place in almost every industry^30^, resulting in dynamic changes of dependency groups in financial networks. In social networks, each individual reacts adaptively to its own changing propensities and capabilities, which leads to dynamical dependency groups^31^. In epidemiological networks, due to more and more frequent social personnel flow, accidentally coming in contact with others gives rise to time-dependent dependency groups. Therefore, it is necessary to explore cascading failures of networks under dynamical dependency.

The dependency property in networks has two sides. It is well known that the dependency property makes these networks more fragile under failure. However, the dependency property contributes to the recovery of failure nodes in dependency groups under certain conditions. In a dependency group, a fraction of nodes exists whose failure has no effect on the function of the group^23^. These failed nodes can be recovered, called dependency recovery, by means of the dependency property. For example, for communities in social networks, the process of social influence can promote convergence in behaviour^32^. Conformity behaviour makes the minority swim with the tide. Recently, network recovery mechanisms, such as targeted recovery^33^, greedy recovery^34^ and spontaneous recovery^35^ after a time period, have been investigated with respect to network robustness. All of these efforts indicate that network robustness is the gaming result between failure and the recovery of nodes, especially for networks with large time scale evolution, e.g., social and economic systems^17^. Therefore, the failure and recovery of dynamical dependency groups coexist in a variety of complex networks, inspiring us to explore how robust or vulnerable these networks behave.

The aim of this study is to determine the effect of dynamical dependency groups on the percolation of networks with a recovery mechanism. Our main contribution can be summarized as follows: (I) We simultaneously introduce dynamical dependency groups and the recovery mechanism to investigate the robustness of networks. By means of a general evolving network model, we can understand the cascading failures of real networks better, where varying dependency relation is widespread. Then, we find that the dynamical dependency among nodes results in disintegrating the network in the form of an abrupt phase transition after an initial random failure of nodes. (II) We consider the robustness discussed here as the gaming result between the failure mechanisms and recovery mechanisms of a network. Through our analysis, we find that dependency strength has a nonlinear effect on network robustness, which is significantly different from the linear effect on networks with static dependency. This robustness, reflecting the capacity of real networks against failure, provides insight to the network designer. This article is organized as follows. In the section of results, firstly, we present an evolving network model consisting of failure mechanisms, a recovery mechanism and a dynamic mechanism of dependency groups. Then, we investigate the effects of dependency strength and the size of the dependency group on network robustness. The relevant discussions are given in the next section. By employing generating function techniques, we give analytical results based on our presented model in the methods section.

## Results

### An Evolving Network Model with Dynamical Dependency

We here introduce a generic evolving model with dynamical dependency groups under the coexistence of cascading failure and a recovery mechanism as a basis of our theoretical and numerical analysis. Consider an arbitrary network of size *N* with degree distribution *P*_*k*_. Initially, a fraction *q* of nodes is chosen randomly to form dependency groups with size *g*; all other nodes with the fraction (1 − *q*) do not belong to any dependency groups. After randomly removing a fraction 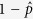 of nodes and their links, the failed nodes may cause the other nodes to disconnect from the network (percolation process^18^). All of the failure nodes are called the initial removal 1 − *p*. Then, dependency groups undergo the dependency process and dependency evolution.

- **Dependency Process.** If the fraction of the failure nodes of a group is equal to or larger than the dependency failure threshold, the group will fail, i.e., all nodes in this group will fail, even though they still connect to the network via connectivity links (dependency failure process^23^). If the fraction of the failure nodes of a group is less than or equal to the dependency recovery threshold, the group will recover, i.e., the failure nodes in this group will go back to normal (dependency recovery process). This dependency process, including the dependency failure process and the dependency recovery process, can be described by the following mathematical model:

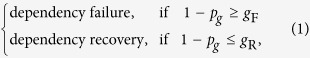


where *p*_*g*_ is the fraction of functional nodes in this group; *g*_F_ and *g*_R_ are the failure threshold and the recovery threshold, respectively.

- **Dependency Evolution.** After one dependency process triggered by failed nodes and its subsequent percolation process, previous dependency groups will evolve into new dependency groups. These new dependency groups still consist of randomly chosen nodes.

Each dependency process and its subsequent percolation process are called one iteration. During one iteration, the failed nodes may trigger more failed nodes, recover partially from failure, or have no effect on other nodes. Therefore, the frequency of group change has no influence on network robustness. If the failed nodes trigger more failed nodes, the frequency only shifts the number of iterations in the cascading process; otherwise, the cascading process stops before the dependency groups change. To facilitate the theoretical analysis, our model assumes that the new dependency groups will replace previous dependency groups between each iteration. Once the cascade process begins, the dependency process and percolation process will occur alternately until there are no further nodes whose state is different from others. Note that under static dependency, our model is equivalent to the failure mechanism proposed by Wang *et al*.^23^ when *gg*_R_ < 1; furthermore, when *gg*_R_ < 1 and *gg*_F_ < 1, our model reduces to the representative failure mechanism proposed by Parshani *et al*.^18^.

**Figure 1:**
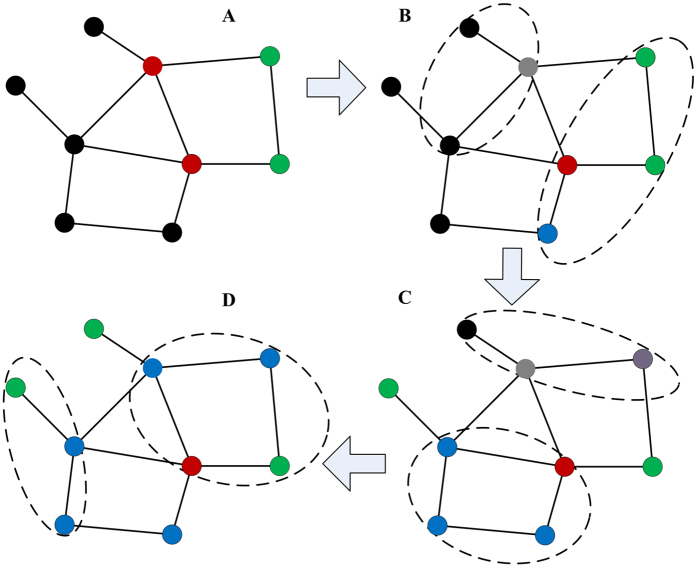
Network with dynamical dependency group. The network is composed of nine nodes, eleven connectivity links (**A**), and two dependency groups (dashed lines). The relation among the nodes is time-dependent in every cascading process, including a dependency process and a percolation process. In each group, if a fraction *g*_F_ or more of nodes fail, the group will fail, i.e., all of the nodes of this group fail. If no more than a fraction *g*_R_ of nodes fail, the group will recover, that is, the failing nodes in this group will go back to normal. *g*_F_ = 0.5 and *g*_R_ = 0.4 are taken as an example for this network. (**A**) The red nodes represent the initial random removal. The nodes removed due to the percolation process are marked in green. (**B**) Due to the dependency process, the nodes removed are marked in blue and the nodes recovered are marked in grey. (**C**,**D**) Due to dynamical dependency, the dependency process and percolation process result in change of the node’s state again. Each graph shows the state after one cascading process.

**Figure 2:**
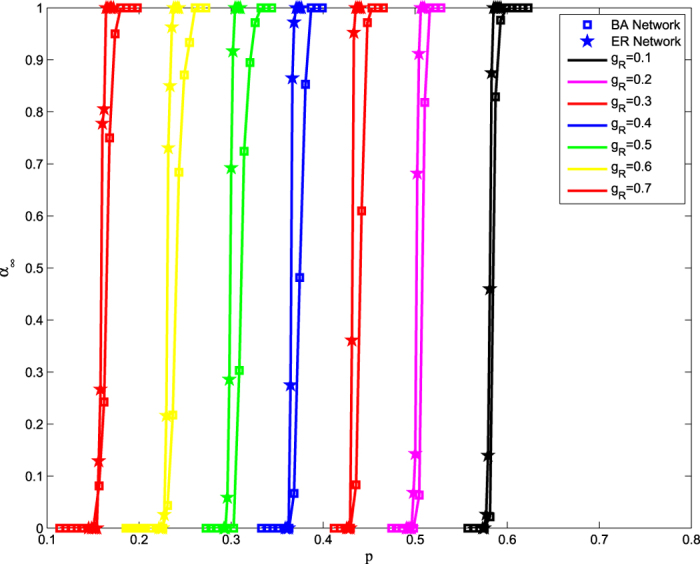
The simulation results of an ER network and BA network for different *g*_R_. In simulations, the parameters are set as: *N* = 2 × 10^4^, *k* = 10, *g* = 10, *q* = 1, *g*_F_ = 0.8. Simulation results show the abrupt phase transitions in the ER and BA networks. The fraction of nodes in the giant component at the end of the cascading process, *α*_∞_, is shown as a function of *p*. Note that in our model, the final state of the giant component is 0–1 binary due to the dynamical dependency. Therefore, the value of *α*_∞_ is not the real size of the giant component of the steady state, but rather, the average of *α*_∞_ for several realizations of computer simulations.

To illustrate our model mentioned above explicitly, we give a typical simple example, as shown in Fig. 1. We find that, by means of simulations, an initial failure of network nodes disintegrates the network in the form of an abrupt transition if the dynamical dependency always exists. The property, i.e., that the cascading process leads to an abrupt transition, exists for a wide range of topologies, including Erdős-Rényi (ER) and Barabási-Albert scale free (BA) networks, indicating that it is a general property of many networks (Fig. 2). This can be explained qualitatively by our model. Those networks with a small fraction of removed nodes will recover from failure. Conversely, due to a large fraction of removed nodes, the entire network will be fragmented.

### Gaming between Failure and Recovery

In reality, how to improve network robustness or protect a network from vulnerability remains an overarching concern. From Fig. 2, we can see that networks with a larger recovery threshold are more robust, while a smaller failure threshold makes the network more vulnerable. It seems good to strengthen robustness by increasing the recovery failure threshold and the failure threshold. However, we cannot continuously increase the recovery threshold and the failure threshold of dependency groups to enhance robustness due to the cost constraint. Under a certain recovery threshold and failure threshold, we pay more attention to identifying the network property with which we can improve network robustness against random failure. Through our analysis in the Methods section, we find that the dependency strength and size of the dependency group play important roles in the cascading process. The evolving behaviour of a network to cascading failure is affected significantly when these parameters are changed. Hence, we investigate the effects of the dependency strength and size of the dependency group on the network robustness.

**Figure 3:**
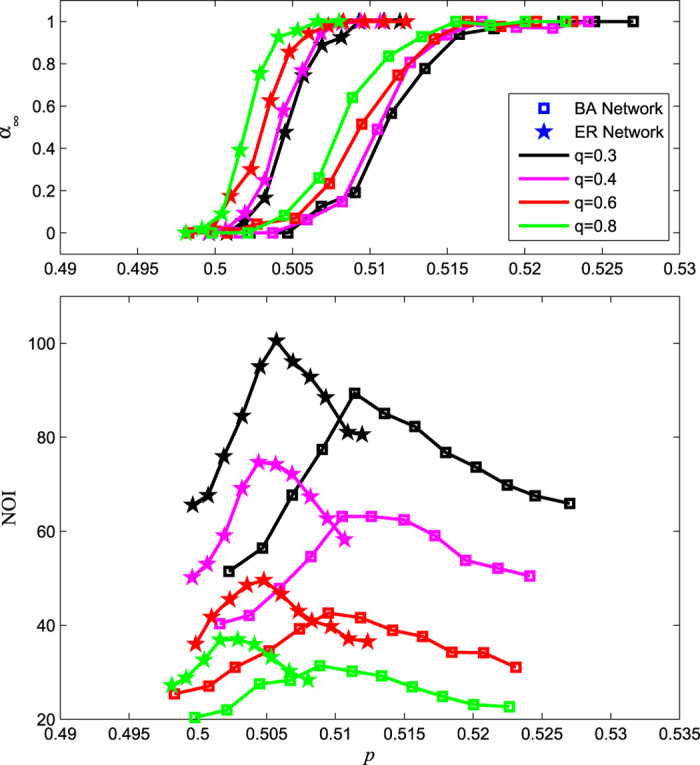
Effects of different dependency strengths *q* on ER and BA networks. In simulations, the sizes of the ER network (pentagram) and BA network (square) are both set as 2 × 10^4^, the average degree is 10, the failure threshold *g*_F_ = 0.8, and the recovery threshold *g*_R_ = 0.2. The simulation results show the size of giant component *α*_∞_ and the number of iterative failures (NOI) of networks under different dependency strengths *q* = 0.3, *q* = 0.4, *q* = 0.6, *q* = 0.8. Note that the value of *α*_∞_ is the average of *α*_∞_ for several realizations of computer simulations.

Regarding the dependency strength of a network, previous studies^18^,^26^ have demonstrated that the dependency strength *q* has a critical effect on the robustness of networks: reducing the dependency strength results in a change from a first-order to a second-order percolation transition. Nevertheless, in our model, the existence of dynamical dependency among nodes can lead to an abrupt percolation phase transition. This is because the synergy between the cascading failure process and the cascading recovery process will result in failure or recovery of the whole network when reaching a steady state. Moreover, it is observed that the dependency strength accelerates the cascading process of networks. Figure 3 shows the fraction of giant component at the end of cascading process *α*_∞_ and the number of iterative failures (NOI) of the same networks under different dependency strength. The networks include the ER network (pentagram) and BA network (square). With decreasing dependency strength *q*, the NOI increases significantly. This can be explained by Eq. (5) in the Methods section; when we reduce the dependency strength *q, α*_∞_ will depend less on the dependency part, which will delay the breakdown time. Furthermore, from Eq. (12), we reveal the nonlinear effect of dependency strength on network robustness.

Next, we study how the size of the dependency group affects the network robustness. According to Eq. (12), the critical point *p*_*c*_ is dependent on the size *g* of the dependency group, the recovery threshold *g*_R_, the failure threshold *g*_F_ and the dependency strength *q*. We all know that the cumulative probability density function of binomial distribution is defined as:


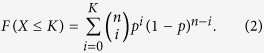


It is an increasing function of probability *p* that increases with the total number of samples *n* if *K* > *np*, while it decreases with *n* if *K* < *np*. Therefore, the critical point *p*_*c*_ increases with the size *g* of the dependency group if


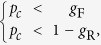


and decreases with *g* if


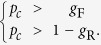


Furthermore, for cases under the situation *p*_*c*_ > *g*_F_ and *p*_*c*_ < 1 − *g*_R_, the condition *g*_F_ < 1 − *g*_R_ is always met. Thus, we can obtain that *p*_*c*_ decreases with *g* when:


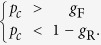


In real networks, there are no situations of *p*_*c*_ < *g*_F_ and *p*_*c*_ > 1 − *g*_R_ because, in general, *g*_F_ > *g*_R_.

**Figure 4:**
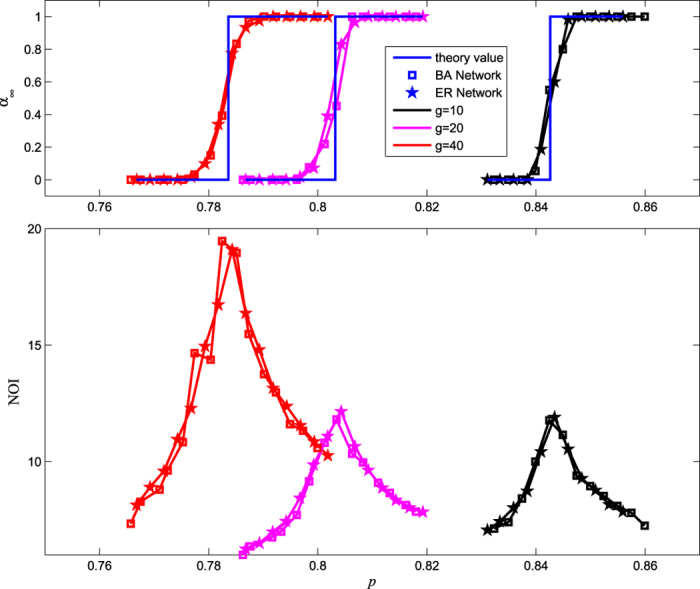
The critical point *p*_*c*_ of the case versus *g*. The parameters are set as: *k* = 10, *q* = 1, *g*_F_ = 0.4 and *g*_R_ = 0.1. (**a**) The size of the giant component of steady state *α*_∞_ versus *p*. The pentagram symbol (ER network) and square symbol (BA network) represent simulation results, while the blue solid lines show the corresponding analytical prediction of Eq. (12). (**b**) As noted in ref. 18, the number of iterative failures (NOI) sharply increases when *p* approaches the critical point; thus the sharp peaks can identify the corresponding critical points *p*_*c*_ for the abrupt transition region. Moreover, the peak of NOI increases with the size of the group, which indicates that there are longer times to recover for fault systems. Note that the value of *α*_∞_ is the average of *α*_∞_ for several realizations of computer simulations.

Roughly speaking, networks with a larger dependency group size are more robust if *p*_*c*_ < *g*_F_ and more vulnerable if *p*_*c*_ > *g*_F_. Simulation results show the validity of our analysis. In Fig. 4, we plot the values of *p*_*c*_ of different size *g* of an ER network (pentagram) and BA network (square) for a given recovery threshold (0.1) and failure threshold (0.4). In these networks, because *p*_*c*_ > *g*_F_, *p*_*c*_ decreases with size *g*. This is consistent with the simulation results. Moreover, it has been shown from Fig. 4 that a network with a larger size of dependent groups will more slowly respond to initial removal, which can be reflected by the special behaviour characterizing the NOI in the cascading process. For a larger size of dependent groups, the response approaches steady state much more slowly. This is because the interval of *g*_R_ < 1 − *p* < *g*_F_ increases with the size *g* of the dependency group, that is, the fraction of the network without dependency groups, which equivalently reduces the dependency strength.

## Discussion

Concerning the dynamical dependency in real networks, we propose a cascading failure model for a network with dynamical dependency groups and a recovery mechanism. In our presented evolving network model, the dependency relation among nodes is time-varying, which is more accordant with real networks, and the failing node could be recovered if the group it belongs to satisfies the recovery condition. Our presented model is more universal because it covers the model in which the failure of one or some nodes can lead to the failure of a dependency group when *gg*_R_ < 1.

Both simulation and analytical results reveal that the network disintegrates in the form of an abrupt transition. According to analysis, the critical point of our model depends on the size of the dependent group, the dependency strength, the recovery threshold and the failure threshold. Furthermore, we find that, in general, a larger dependency group always makes such a network more vulnerable when the failure threshold *g*_F_ < *p*_*c*_, whereas for the failure threshold *g*_F_ > *p*_*c*_, a network with a larger dependency group is more vulnerable. No matter the number of failure and recovery thresholds, a larger group size gives the network more power to resist the initial removal, which indicates that we have more time to recover the failure nodes before the network breaks down. By means of our evolving network model and the analysis, we can have a better understanding of the robustness of a real networked system with the dependency property.

## Methods

To solve this model, the exact analytical solution of the dependency process and the percolation process can be obtained by means of the mean-field approximation and the generating function techniques, respectively. In a mean-field approximation, after initially removing a fraction 1 − *p* of nodes, 1 − *p* is the average probability that any node has failed. Let *P*_R_ and *P*_F_ be the probability that a dependency group is functioning and failing, respectively. As the setting of our model for the typical case of *q* = 1, *P*_R_ and *P*_F_ can be obtained according to Eq. (1):


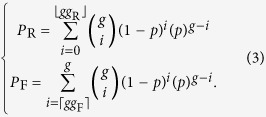


where 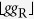 is the largest integer smaller than or equal to *gg*_R_; 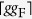 is the smallest integer larger than or equal to *gg*_F_.

Thus, the fraction of nodes that remain functional after the dependency process, *g*_D_(*p*), is:


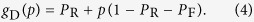


Following a similar approach for the general case of 0 ≤ *q* ≤ 1 yields the universal function *g*_D_(*p*):


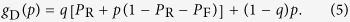


Analogous to *g*_D_(*p*), *g*_P_(*p*) is defined as the fraction of nodes belonging to the giant component of the connectivity network after random removal of fraction 1 − *p* of the nodes. The percolation process can be solved analytically by using the apparatus of generating functions^21^,^36^,^37^. As shown in the generating function methods, we introduce the generating function of the degree distributions *G*_0_(*ξ*) = ∑_*k*_ *P(k)ξ*^*k*^ and the generating function of the underlining branching processes 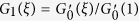. Random removal of 1 − *p* nodes will change the degree distribution of the remaining nodes; then, the generating function of the new distribution is equal to the generating function of the original distribution, with the argument equal to 1 − *p*(1 − *ξ*)^36^. The fraction of nodes that belongs to the giant component after the removal of 1 − *p* nodes is ref. 37


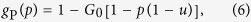


where *u* = *u(p*) satisfies the self-consistency relation


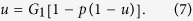


It follows from the previous analysis that the network will return back to normal if the number of nodes recovering from failure, Δ_TR_, is larger than the number of nodes failing, Δ_TF_, at every iteration; otherwise, if Δ_TR_ < Δ_TF_, the network will undergo crash. Therefore, it is natural to pay more attention to the critical point *p*_*c*_ such that:


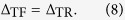


Because functions *g*_D_(*p*) and *g*_P_(*p*) are non-decreasing, we can find the critical point of this model at the first iteration. For the first iteration, the number of recovery nodes Δ_TR_ is:


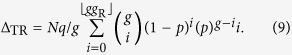


The number of failed nodes in the dependency process, Δ_TDF_, is:


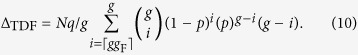


After the dependency process, there may be several nodes that need to be removed in the percolation process. Before this percolation process, the accumulated failure, including the initial removal of (1 − *p*) and the removal of (Δ_TR_ − Δ_TF_)/*N* due to the dependency process, is equivalent to a single random removal of [1 − *p*_0_ − (Δ_TR_ − Δ_TF_)/*N*], where *p*_0_ = *p*/(1 − *u*), *u* = *G*_1_[1 − *p*_0_(1 − *u*)]. Therefore, the number of failing nodes in the first cascading process, Δ_TF_, is:


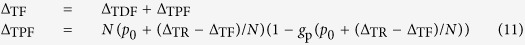


According to Eq. (8), the critical point of this model can be obtained. Note that Δ_TF_ or Δ_TR_ is not the real value of every dependency group, just a mean value of all groups. Therefore, phase transition occurs at around the critical point for real networks.

The formalism presented above is generic and applicable to any random network with arbitrary degree distribution. In the case of a common ER random network whose degrees are Poisson distributed^38^,^39^, the problem can be solved explicitly. Let the average degree of the network be *k*. Then, the generating function can be written as *G*_1_(*ξ*) = *G*_0_(*ξ*) = exp [*k(ξ* − 1)]. Substituting these generating functions into Eqs (6) and (7), we derive *g*_P_(*p*) = 1 − *u* and *u* = exp (*kp(u* − 1)), respectively. Thus, Δ_TPF_ = *Np*_1_*u*, where *p*_1_ = *p*_0_ + (Δ_TR_ − Δ_TF_)/*N* and *u* = exp (*kp*_1_(*u* − 1)). Furthermore, we get the following equations for the critical point *p*_*c*_:


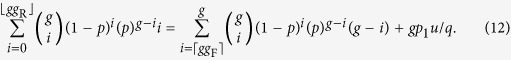


**Figure 5:**
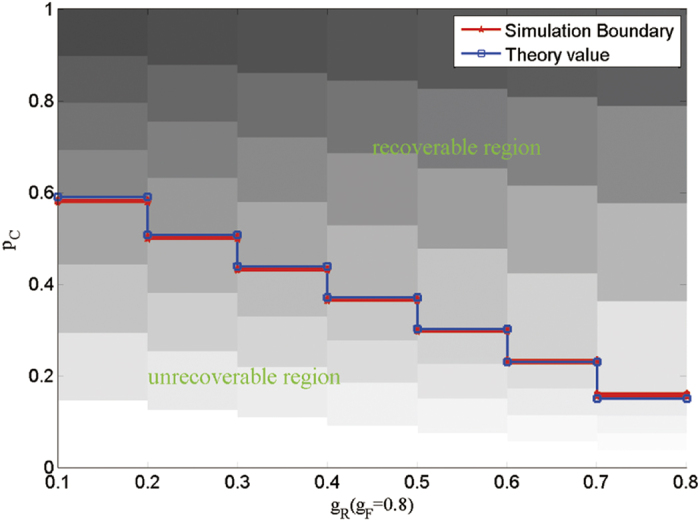
Comparison between simulations and theoretical results of critical point *p*_*c*_ versus *g*_R_. The results are shown for an ER network with *k* = 10, *g* = 10, *q* = 1, and *g*_F_ = 0.8. The theoretical results (redline) are calculated according to Eq. (12) and compared to several realizations of computer simulations for networks of size *N* = 2 × 10^4^.

Equation (12) gives the relation of the critical point *p*_*c*_, the failure threshold *g*_F_, the recovery threshold *g*_R_ and the dependency strength *q*. To validate the theoretical results, we carry out simulations on an ER network of size *N* = 2 × 10^4^ and plot the size of the giant component at the end of the cascading process as a function of the recovery threshold *g*_R_ in Fig. 5. It is observed from Fig. 5 that the simulation results agree well with the theoretical results.

## Additional Information

**How to cite this article**: Bai, Y.-N. *et al*. Robustness and Vulnerability of Networks with Dynamical Dependency Groups. *Sci. Rep.* **6**, 37749; doi: 10.1038/srep37749 (2016).

**Publisher's note:** Springer Nature remains neutral with regard to jurisdictional claims in published maps and institutional affiliations.
